# Feeding a Diet Enriched in Docosahexaenoic Acid to Lactating Dams Improves the Tolerance Response to Egg Protein in Suckled Pups

**DOI:** 10.3390/nu8020103

**Published:** 2016-02-19

**Authors:** Caroline Richard, Erin D. Lewis, Susan Goruk, Catherine J. Field

**Affiliations:** Department of Agricultural Food and Nutritional Science, University of Alberta, Edmonton, AB T6G 2E1, Canada; cr5@ualberta.ca (C.R.); edlewis@ualberta.ca (E.D.L.); sgoruk@ualberta.ca (S.G.)

**Keywords:** immunology, suckling period, offspring, development, oral tolerance

## Abstract

The objective of this study was to determine the effect of feeding a maternal diet supplemented with docosahexaenoic acid (DHA) during the suckling period on the development of the immune system and oral tolerance (OT) in offspring. Dams were randomized to consume one of two nutritionally adequate diets throughout the suckling period: control (N = 12, 0% DHA) or DHA (N = 8, 0.9% DHA) diet. At 11 days, pups from each dam were randomly assigned to a mucosal OT challenge: the placebo or the ovalbumin (OVA) treatment. At three weeks, plasma immunoglobulins and splenocyte cytokine production *ex vivo* were measured. OVA-tolerized pups had a lower Th2 (IL-13) response to OVA despite the presence of more activated T cells and memory cells (CD27+, all *p* < 0.05). Feeding a high DHA diet improved the ability of splenocytes to respond to mitogens toward a skewed Th1 response and led to a higher IL-10 and a lower TGF-β production after stimulation with OVA (all *p* < 0.05). Untolerized DHA-fed pups had lower plasma concentrations of OVA-specific immunoglobulin E (*p* for interaction < 0.05). Overall, feeding a high DHA maternal diet improves the tolerance response in untolerized suckled pups in a direction that is thought to be beneficial for the establishment of OT.

## 1. Introduction

An important T cell mediated process that occurs early in life is the development of oral tolerance (OT), referring to the ability to distinguish between harmful and harmless antigens delivered via the oral route [1]. Food allergies are believed to be the result of a failure to develop OT to dietary antigens. At birth, T cells have a lower ability to respond to immune challenges (*i.e.*, proteins, pathogens, toxins) and when they respond, they favor a type 2 T helper (Th2) cytokine response (*i.e.*, IL-4, IL-5, IL-6 and IL-13) over a Th1 response (IL-2, IFN-γ and TNF-α) [2]. It is proposed that this period of immune system “immaturity” renders the infant more vulnerable to exogenous challenges by pathogens [3]. It is also hypothesized that the inappropriate Th2 response increases the risk for allergic diseases and asthma [2]. The effect of maternal and infant diet on this process has not been well established.

The amount and type of long chain polyunsaturated fatty acids (LCPUFAs), including the omega-6 (*n*-6) arachidonic (AA) and the *n*-3 docosahexaenoic (DHA) acid, have both been shown to have a major impact on inflammation and other aspects of immune function in adults (reviewed by [4]). There are also some promising results from studies demonstrating that supplementation or high intakes of *n*-3 LCPUFA during pregnancy, can reduce the risk/symptoms of asthma [5,6,7] and allergies [5] in infants with a history of atopic diseases. These data suggest that *n*-3 LCPUFA delivery during critical modulatory periods such as pregnancy are important in the development of OT. However, very little is known regarding the impact of maternal LCPUFA supplementation during the postnatal period on the immune system development and the establishment of OT. Although there is evidence that OT can be induced at any time in the lifecycle [1], this process typically occurs in humans during infancy and in rodents during the first 21 days of life [8]. This implies that nutritional intervention during the lactation/suckling period could have a major impact on the development of the immune system.

The AA content in immune cells membrane could also be important for tolerance as there is a rapid increase in the content of AA in the thymus in the early postnatal period [9], the most important period of T cell development and tolerance [1]. Feeding diets with a low *n*-6/*n*-3 LCPUFA ratio increased the content of *n*-3 LCPUFA and decreased *n*-6 (particularly AA) in cell membrane phospholipids [9]. However, most studies so far on *n*-3 supplementation have provided very high doses of *n*-3 LCPUFA which in turn lower the *n*-6/*n*-3 LCPUFA ratio of the diet (primarily by reducing cell content of AA) and lead to an overall anti-inflammatory effect [9,10,11,12,13]. While this is expected to be beneficial in adults with chronic low-grade inflammation, it is likely undesirable in the context of immune system development. In support of this, infant formula enriched in AA/DHA has been reported to have beneficial effects on the appearance of atopic symptoms and the development of the immune system compared to unsupplemented formula [14,15]. Nevertheless, the amount and importance of AA in the diet of infants has been recently questioned [16,17,18,19]. 

Importantly, most of the previous work has investigated infants not exposed to breast milk, *i.e.*, formula-fed or early weaned infants [20]. The objective of the current study was therefore to determine the effect of feeding a maternal diet supplemented with DHA, while containing sufficient amounts of AA (so as to achieve the content reported in breast milk), during the suckling period on the development of the immune system and the establishment of OT in offspring. We assessed, in a cross-over design, the development of OT using a healthy rat model in a context of “real life”, where a mucosal OT treatment to ovalbumin (OVA, a dietary antigen) was induced in suckled pups. Immune function and OT were assessed by cytokine production by splenocytes stimulated *ex vivo* with mitogens or OVA and plasma OVA-specific immunoglobulins concentrations.

## 2. Materials and Methods

### 2.1. Animals and Diets

**Figure 1:**
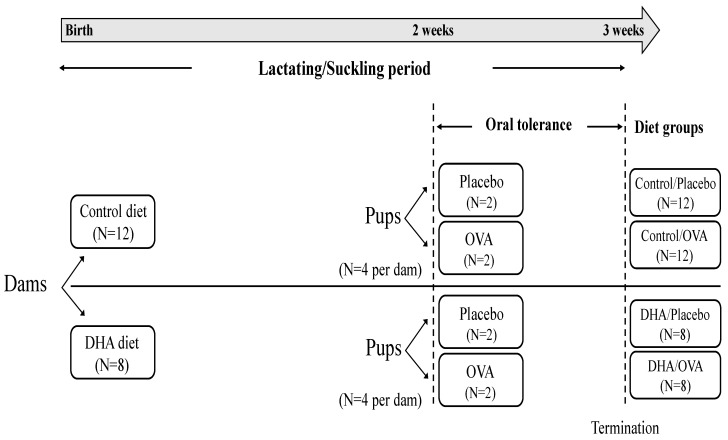
Animal study design.

All animal care and experimental protocols were conducted in accordance with the Canadian Council on Animal Care and approved by the University of Alberta Animal Ethics Committee (AUP00000125). Primiparous Sprague-Dawley rats (*n* = 20) were obtained from Charles River Laboratories (Montreal, QC, Canada) on day 14 of gestation and were individually housed in a temperature and humidity controlled environment with a 12/12-h reversed light cycle. Dams were fed standard rat chow (Lab diet 5001; PMI Nutrition International, Brentwood, MO, USA) throughout gestation, then randomized to one of two nutritionally complete experimental diets 24–48 h prior to parturition: control diet (0.04% AA and 0% w/w DHA, N = 12) or high DHA diet (0.04% AA and 0.18% w/w DHA, N = 8). The litters were culled to 10 pups per dam and diets were fed *ad libitum* throughout lactation. Offspring were kept with their mothers for the duration of the suckling period (21 days). At 11 days, pups from each dam were randomly assigned to a mucosal OT treatment: the placebo (sucrose) or the ovalbumin (OVA) treatment (see immunization protocol below). At 3 weeks, pups were terminated and two pups from the same dam within each oral tolerance treatment were pooled. This study design allowed us to specifically investigate the impact of feeding a high DHA diet during the suckling period in the context where an OT treatment has been induced and resulted in four different diet groups as presented in Figure 1. Each group contains 8 pups for the DHA diet (N = 8 tolerized pups and N = 8 untolerized pups) and 12 pups for the control diet (N = 12 tolerized pups and N = 12 untolerized pups).

Both experimental diets were isocaloric, isonitrogenous and the macronutrient and micronutrient composition were identical, with the exception of PUFA content as shown in Table 1. The non-lipid nutrient composition of the experimental diets has been previously described [21]. The added fat mixture to the rodent diet was composed of flaxseed oil, sunflower oil, saturated canola oil, olive oil, a high AA oil and a high DHA oil (both AA and DHA oils were provided by DSM (Nutritional Products, Columbia, Maryland, US)) and fatty acids were matched closely so the diet primarily differed only in the total omega-3 content. Both diets met the essential fatty acid requirements of the rodent and had similar PUFA/saturated fatty acid ratio. Diets were prepared weekly and stored at 4 °C until fed; feed cups were replaced every 2–3 days to prevent oxidation. Dietary intake and body weights were monitored regularly throughout the intervention. 

Dams were randomly assigned to the control diet (N = 12) or the high DHA diet (N = 8) for the duration of the lactating/suckling period (3 weeks). At two weeks of age, 4 pups from each dam were randomly assigned to a mucosal oral tolerance (OT) treatment: placebo (sucrose, N = 2 per dam) or ovalbumin (OVA, N = 2 per dam) in a cross over design. At the end of the suckling period, pups were terminated and pooled according to their respective dam/OT treatment. Since the dams are the experimental unit in this study design the number of observations within each group is equal to the number of dams.

### 2.2. Ovalbumin Administration and Immunization

In order to induce mucosal oral tolerance (OT) against OVA (Sigma-Aldrich Canada Ltd. Oakville, ON, Canada), pups from each litter were orally fed 100 µL of sterile filtered OVA or placebo (sucrose; Fisher Scientific). OVA (8 mg/mL in 8% sucrose solution) and placebo (8% in water) solutions were delivered orally via syringe once daily for 5 days (from 11 to 15 days of age). Twenty-four hours prior to termination, all pups received an intraperitoneal (IP) injection of 10 µg ovalbumin in 100 µL PBS to induce acute sensitization to the allergen. As established by Odemuyiwa *et al.* [23] an IP injection of 10 µg of OVA is sufficient to induce allergic sensitization. Our aim was to assess the development of oral tolerance using a healthy rat model in a context of “real life”, in suckled animals. In human, the suckling period generally last a year and food are first introduced around 4–6 months, despite current recommendation for exclusive breast feeding to 6–8 months of age. The first 21 days in rat corresponds roughly to the first year of life in human and we started the immunization protocol at 11 days.

### 2.3. Tissue Collection

At day 21 after birth, pups were euthanized by CO_2_ asphyxiation and subsequent cervical dislocation. Spleens and mesenteric lymph nodes were collected aseptically and immune cells were isolated (see below). Livers were collected aseptically, weighed, snap frozen in liquid nitrogen and stored at −80 °C until analysis.

### 2.4. Immune Cell Isolation

Immune cells were isolated from spleens and mesenteric lymph nodes (MLN) as previously described [24]. Briefly, single cell suspensions were obtained by disrupting tissue through a nylon mesh screen in sterile Krebs–Ringer HEPES buffer with bovine serum albumin (5 g/L) (Sigma-Aldrich Canada Ltd., Oakville, ON, Canada). Erythrocytes were lysed with ammonium chloride lysis buffer (155 mM NH_4_Cl, 0.1 mM EDTA, 10 mM KHCO_3_; Fisher Scientific, Edmonton, AB, Canada). Cells were washed and re-suspended in complete culture medium (RPMI 1640 medium supplemented with 5% (v/v) heat-inactivated fetal calf serum, 25 mM HEPES, 2.5 mM 2-mercaptoethanol and 1% antibiotic/antimycotic (pH 7.4); Invitrogen, Burlington, ON, Canada). Cells were counted on a haemocytometer using trypan blue dye (Sigma) exclusion and diluted to 1.25 × 10^6^ cells/mL. 

### 2.5. Immune Cell Phenotype Analysis

Immune cell subsets in freshly isolated splenocytes were identified by direct immunofluorescence assay as previously described [15]. Briefly, immune cells (200,000) were incubated for 30 min at 4 °C with pre-labelled monoclonal antibodies applied in combination to quantify various immune cell phenotypes. Four-color flow cytometry allowed determination of the following surface molecule combinations for spleen: CD28/CD3/CD8/CD4, CD28/CD152/CD8/CD4, CD25/CD152/CD8/CD4, CD25/CD127/CD8/CD4, CD27/CD8/CD4, CD27/OX12/OX6/CD45ra, CD71/CD8/CD4, OX12/OX6/CD80, CD86/CD80/CD45ra, CD68/CD284/CD11b/c, OX62/CD25/OX6, CD161/OX62/CD3, IgG/IgM, IgA; and for MLN lymphocytes: CD28/CD3/CD8/CD4, CD25/CD152/CD8/CD4, CD25/CD127/CD8/CD4 and CD27/CD8/CD4. All antibodies were purchased from eBiosciences (San Diego, CA, USA) or BD Biosciences (Mississauga, ON, Canada). OX-62 is the rodent alpha E2 integrin which is expressed by dendritic cells, OX12 is the Ig light chain of rodent B cells and OX6 or CD74 is the major histocompatibility complex class II. Cells were then washed and fixed in paraformaldehyde (10 g/L; Anachemia Science, Montreal, PQ, Canada) in phosphate-buffered saline (PBS) with sodium azide as the preservative. Immune cells were all acquired within 72 h by flow cytometry (FACSCalibur; Becton Dickinson, San Jose, CA, USA) according to the relative fluorescence intensity using Kaluza Software (Beckman Coulter, Mississauga, ON, Canana). 

### 2.6. Measurements of *Ex Vivo* Cytokine Secretion by Mitogen-Stimulated Splenocytes and Plasma Ova-Specific Immunoglobulin Concentrations

Cytokine production by splenocytes was measured as previously described [25]. Briefly, immune cells (1.25 × 10^6^ cells/mL) were cultured for 48 h without mitogen (unstimulated cells) or with concanavalin A (ConA, 2.5 µg/mL; MP Biomedicals, Montreal, Quebec, Canada), lipopolysaccharide (LPS, 100 µg/mL, Sigma), or ovalbumin (OVA, 100 µg/mL, Sigma). Cells were then centrifuged for 10 min at 1000 rpm and the supernatants kept at −80 °C. Commercial ELISA kits were used to measure the concentrations of IL-1β, IL-2, IL-6, IL-10, TNF-α, TGF-β, and IFN-γ according to the manufacturer’s instructions and as described previously [25]. All detection limits were 15.63–4000 pg/mL except for IFN-γ which was 9.76–2500 pg/mL (R&D systems, Minneapolis, MN, USA). IL-13 and IL-5 was measured by sandwich ELISA commercial kits from MyBioSource (supplied by Cedarlane) as per detailed instructions. The detection range of both kits was 15.625 pg/mL–1000 pg/mL). Plasma concentrations of IgG and IgE ova-specific were measured according to the manufacturer’s instructions with a dilution 1:20 for both kits (Alpha Diagnostics Intl. supplied by Cedarlane). Absorbance was read on spectrophotometer and concentrations were calculated from the standard curve (SpectraMax 190, Molecular Devices Sunnyvale, CA, USA) with all measurements were conducted in duplicate with CV < 10%.

### 2.7. Statistical Analysis

Data is reported as mean ± standard error of the mean (SEM) unless indicated otherwise. The study was powered to assess significant changes in immune function (as the primary outcome). Data was analyzed using the PROC MIXED procedure for repeated measures in SAS (v9.4, Cary, NC, USA) with diet and the OT treatment as the main effect (*i.e*., dams’ suckling period diet, the OT treatment and their interaction). In cases where heterogeneous variation was suspected, models assuming either heterogeneous or homogeneous variances were compared based on Akaike Information Criteria (AIC) and the best fitting model was selected. Differences at *p* ≤ 0.05 (two-sided) were considered significant. 

## 3. Results

### 3.1. Growth Parameters and Plasma OVA Specific Immunoglobulin Concentrations

**Figure 2:**
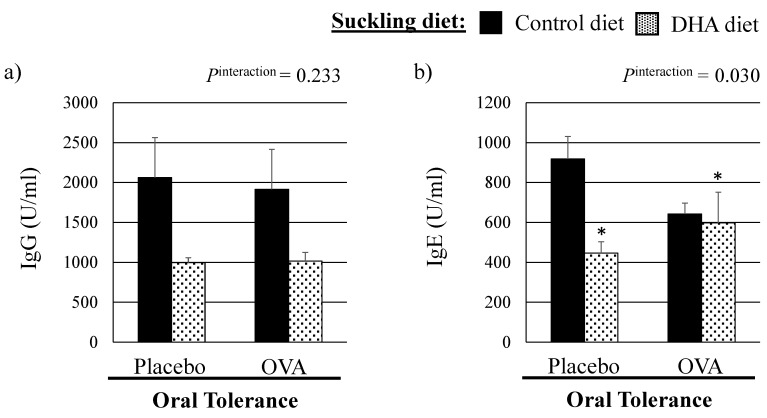
Plasma ova-specific immunoglobulins concentrations in three-week pups according to the suckling maternal diet and the mucosal oral tolerance treatment. (Ig, immunoglobulin; * Statistically significant effect of the suckling diet (DHA *vs.* control diet), *p* < 0.05. *P*^interaction^, interaction between the suckling diet and the oral tolerance treatment in the Mixed model.).

There was no effect of the suckling diet nor of the OT treatment on final body weight (mean 50.6 ± 2.3 g), spleen weight (mean 0.28 ± 0.02 g), liver weight (mean 2.25 ± 0.09 g) or gut length (mean 70.2 ± 1.6 cm) of the 21d old pups (Supplementary Table S1). No differences were observed in the number of splenocytes relative to spleen weight between diet groups or OT treatment groups in pups at three weeks. There were no overall significant effects of the OT treatment (*p* = 0.789) or the maternal suckling diet on plasma OVA-specific IgG concentrations, although there was a trend towards a lower OVA-specific IgG concentration in pups from the DHA-fed dams (*p* = 0.102) compared to the control diet (Figure 2). Feeding a maternal diet high in DHA significantly reduced plasma ova-specific IgE concentrations compared to the control diet (*p* = 0.028). However, there was a significant interaction between the maternal suckling diet and the OT treatment (*p* for interaction = 0.030), for which only untolerized pups from dam fed the control diet had meaningful higher plasma ova-specific IgE concentrations.

### 3.2. Ex Vivo Cytokine Production by Mitogen-Stimulated Splenocytes 

**Figure 3:**
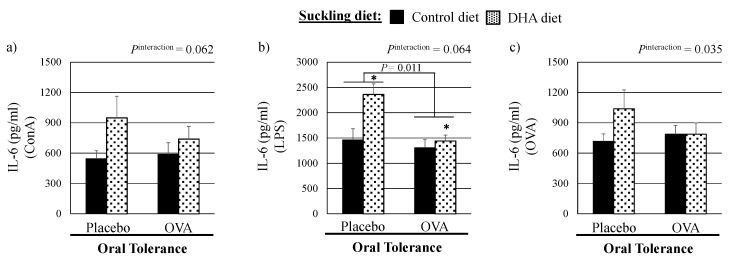
Interleukin-6 production in three-week pups in response to mitogens and ovalbumin stimulation according to the suckling maternal diet and the mucosal oral tolerance treatment. (ConA, concanavalin; DHA, docosahexaenoic acid; IL, interleukin; LPS, lipopolysaccharide; OVA, ovalbumin; * Statistically significant effect of the suckling diet (DHA *vs.* control diet), *p* < 0.05. *p* < 0.05, statistically significant effect of the oral tolerance treatment (placebo *vs.* OVA). *P*^interaction^, interaction between the suckling diet and the oral tolerance treatment in the Mixed model.).

Con A (a T cell mitogen): Regardless of the OT treatment, pups from dams fed the high DHA diet during the suckling period produced significantly more IFN-γ (*p* = 0.045) compared with the control diet (Table 2). There was a trend towards a higher production of IL-6 with the DHA diet in untolerized pups (interaction *p* = 0.062, Figure 3a). Feeding a high DHA diet during the suckling period had no impact on the production of IL-2, IL-10 and TNF-α by ConA-stimulated splenocytes (Table 2).

LPS (a B and antigen-presenting cells stimulant): After LPS stimulation, splenocytes from pups who received the high DHA diet during the suckling period produced more IL-6 (trend that this occurred only the untolerized pups (interaction *p* = 0.064)), IL-10 and TNF-α compared to pups who received the control diet (all *p* < 0.05). Tolerized pups produced less IL-6 (*p* = 0.011) compared to untolerized pups (Figure 3b). The production of IL-1β and IFN-γ by splenocytes stimulated with LPS did not differ significantly amongst groups.

OVA (dietary antigen): Following stimulation with OVA, tolerized pups produced less IL-13 (*p* < 0.001) compared to untolerized pups. IL-2 production was similar amongst groups, but pups from dams fed the high DHA diet produced more IL-10 and less TGF-β after OVA stimulation (both *p* < 0.02) compared with pups who received the control diet. In untolerized pups, feeding the DHA diet resulted in a higher production of IL-6 (interaction *p* < 0.035, Figure 3c). IL-5 was only measured in the supernatant of OVA-stimulated splenocytes and was undetectable.

### 3.3. Immune Cell Phenotypes in Spleen

Overall, there was no effect of feeding a diet high in DHA during the suckling period on pups’ spleen immune cell distributions (Table 3). However, pups who received the OVA OT treatment had a small (6.3%) but significant higher proportion of total T cells (CD3+) compared to pups who received the placebo OT treatment which was attributed to a higher proportion of cytotoxic T cells (CD3+CD8+, both *p* < 0.01). Compared to the placebo treatment, the OT treatment with OVA also resulted in a higher proportion of total cells expressing the co-stimulatory molecule (CD28) along with a higher proportion of cytotoxic T cells expressing CD28 (% of CD3+CD8+ also expressing CD28+, both *p* < 0.02). A higher proportion of total cells expressing OX62 (the alpha E^2^ integrin) and CD3+OX62+ cells was observed in pups who received the OVA OT treatment *vs.* the placebo treatment (both *p* < 0.02). However, no change was observed in the proportion of dentritic cells (OX62+CD74+ cells). Finally, when pups received the OVA OT treatment, there was a higher proportion of total cells expressing CD27 (*p* = 0.003) compared to those who received the placebo treatment, which was mainly attributed to the subset of OX12+CD27+ cells (although it did not reach statistical significance, *p* = 0.108).

### 3.4. Immune Cell Phenotypes in MLN

Although there was no significant effect of the suckling diet nor the OT treatment on the proportion of total T cells (CD3+), pups who received the OVA OT treatment, similar to what we observed in spleen, had a significant higher proportion of total cells expressing CD8 (*p* = 0.011, Supplementary Table S2) and a trend toward an increase in cytotoxic T cells (CD3+CD8+, *p* = 0.059) compared to pups who received the placebo OT treatment. As a result, the ratio of CD4+/CD8+ was slightly lower in pups that received the mucosal OVA OT treatment compared with the placebo treatment (*p* = 0.019). Compared to the placebo treatment, the OT treatment with OVA resulted in a higher proportion of CD8+CD28+ cells (*p* = 0.034). There was also a significant effect of the suckling diet in which pups from dams fed the high DHA diet had a lower proportion of CD8+CD28+ cells compared to pups who received the control diet (*p* = 0.014).

## 4. Discussion

**Figure 4:**
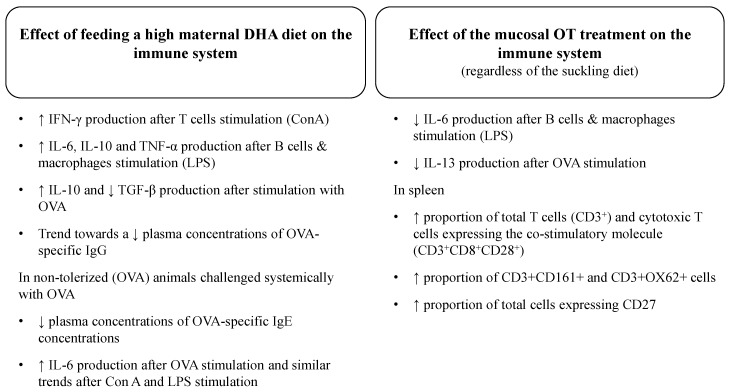
Summary of the effects of feeding a high DHA maternal diet during the suckling period and the mucosal oral tolerance treatment on the development of the immune system in offspring. (ConA, Concanavalin A; DHA, docosahexaenoic acid; IFN-γ, interferon-gamma; Ig, immunoglobulin; IL, interleukin; LPS, lipopolysaccharide; OT, oral tolerance; OVA, ovalbumin; TGF-β, transforming growth factor-beta; TNF-α, tumor necrosis factor-alpha).

We investigated for the first time the effect of feeding a high DHA diet during the suckling period in a cross-over design where a mucosal OT treatment (OVA *vs.* placebo (sucrose)) had been induced in three-week pups on the ability of immune cells to respond to different challenges. We have previously reported that feeding a high DHA diet to lactating dams significantly increased the omega-3 content (mainly 18:3*n*-3 (α-linolenic acid (ALA)), 22:5*n*-3 (DPA) and 22:6*n*-3 (DHA)) of breast milk while slightly increased the AA content as well compared with the control diet, confirming that during the suckling period, pups from dams fed the high DHA diet had a higher intake of DHA (1.09% *vs.* 0.24% of fat) and AA (0.69% *vs.* 0.44% of fat) [22]. Although there was a slight difference in the ALA content of the diet (1.6%), we have previously shown that the ALA composition of splenocyte phospholipids did not differ between suckled pups from dam fed the control diet (0.46% ± 0.01% w/w of total fatty acids) and fed the DHA diet (0.47% ± 0.02% w/w of total fatty acids) [22]. Moreover, we showed that splenocyte phospholipids from pups who received the DHA diet had a higher relative proportion of DHA (1.50% ± 0.18% w/w of total fatty acids) compared with pups from dams fed the control diet (0.64% ± 0.06% w/w of total fatty acids). Consistent with our previous study [22] and others [15,26,27,28], feeding a high DHA diet during suckling had no effect on pups’ growth at three weeks. A summary of the significant effect of feeding a maternal diet enriched in DHA during the suckling period and the mucosal OT treatment on the development of the immune system is presented in Figure 4.

### 4.1. Effect of a High DHA Maternal Diet on the Ex Vivo Cytokine Response to Mitogens in Suckled Pups

We demonstrated that, regardless of whether pups had been orally tolerized to ovalbumin, feeding a maternal diet enriched in DHA while containing sufficient amount of AA during suckling, improved the ability of splenocytes to produce IFN-γ after stimulation with a T cells mitogen (ConA). This is to some extent consistent, with a study conducted by D’Vaz *et al.* in which a daily supplement of fish oil (110 mg EPA and 280 mg DHA) from birth to six months of age in term infants at high risk of atopic diseases led to a higher IFN-γ production in response to phytohaemaglutinin (PHA, another T cell mitogen) [29]. Lauritzen *et al.* have also reported a programming effect on the ability of whole blood cells to produce IFN-γ after stimulation with LPS (a macrophage and B cell mitogen) at 2.5 years in breast-fed infants from mothers supplemented with fish oil during the first four months of lactation [30]. Feeding infant formula enriched in AA/DHA also resulted in a higher IFN-γ production after stimulation with β-lactoglobulin compared with unsupplemented formula [28]. At birth, T cells have a lower ability to respond to immune challenges and when they respond they favor a type 2 T helper (Th2) cytokine response (*i.e.*, IL-4, IL-5, IL-6 and IL-13) over a Th1 response (IL-2, IFN-γ and TNF-α) [2,31]. IFN-g is a key Th1 immunoregulatory cytokine known to inhibit differentiation of Th2 cells and thought to be involved in the development of oral tolerance [32]. Indeed, cord blood cells from infants at low risk for allergy, were found to produce a significantly greater amount of IFN-g than cells from a high-risk group [33]. Therefore, DHA supplementation in the maternal diet during the suckling period beneficially affects immune maturation toward an improved Th1 response, which may have a favorable effect on the establishment of OT early in life.

We also showed that feeding a maternal diet enriched in DHA during the suckling period improved the ability of antigen-presenting cells to produce Th1 but also Th2 type cytokines after challenge. Suckled pups from dam fed the high DHA diet had a higher production of IL-6 and TNF-α after LPS stimulation. Although there was a trend toward an increase production of IL-6 after ConA stimulation in DHA-fed pups, a borderline significant interaction was also observed between the maternal diet and the OT treatment for which only untolerized pups that received the DHA diet had a higher IL-6 production. Individuals with allergic/atopic diseases have an exaggerated response to allergens leading to the production of specific IgE antibodies [34], which have been proposed to involve a dysregulation in the Th1/Th2 balance [35]. TNF-α has been involved in the activation of T cells and the promotion of oral tolerance [33] and reduced production of TNF-α has been observed in allergic infants or those at high risk of developing allergy after stimulation with food allergens [36,37]. IL-6 has been shown to differentially affect the Th1/Th2 balance by promoting Th2 differentiation and also inhibiting Th1 polarization [38]. Since lower plasma concentrations of OVA-specific IgE and a trend towards reduced plasma OVA-specific IgG concentrations were observed in suckled pups that received the high DHA diet, our results suggest that the overall effect of feeding a DHA diet during suckling on the Th1 and Th2 balance was toward a higher Th1 response (including the higher IFN-γ production after ConA). The lower plasma OVA-specific IgE and IgG concentrations also suggests a potential beneficial effect of maternal DHA supplementation during lactation on the establishment of OT.

### 4.2. Effect of the Mucosal Oral Tolerance Treatment on The Immune System

It is often hypothesized that the inappropriate Th2 response increases the risk for allergic diseases and asthma [2]. Splenocytes from OVA-tolerized pups produced significantly less IL-6 after LPS stimulation compared to non-tolerized pups, suggesting a lower Th2 response of antigen-presenting cells in tolerized pups. Moreover, when splenocytes were stimulated with OVA, tolerized pups produced significantly less IL-13 compared with non-tolerized pups. It has been reported that IL-13 can inhibit the production of pro-inflammatory cytokines by monocytes [39] and therefore the lower IL-13 production observed might be partly responsible for the lower IL-6 production. IL-13 is a Th2 cytokine and is thought to be involved in allergic reactions through its role in promoting the release of IgE antibodies from B cells and favoring Th2 cell differentiation [35]. The lower production of IL-13 after *ex vivo* stimulation along with the lower plasma concentrations of OVA-specific IgE (Figure 2B) observed in tolerized pups suggests that tolerance was induced in both the control and the DHA pups that underwent the mucosal immunization process. This is supported by the fact that, irrespective of the maternal diet, the mucosal immunization treatment resulted in an overall more activated T cell population and more memory cells. Pups who had been orally tolerized with OVA had a higher proportion of total T cells which was mainly attributed to a higher proportion of cytotoxic (CD8+) T cells and T cells expressing the co-stimulatory molecule (CD28) and the alpha E^2^ integrin (OX62) isolated from the spleen. Although splenocytes are representative of peripheral immune cells, similar results were observed in immune cells isolated from the MLN for which there was a higher proportion of total cells expressing CD8 and a trend toward a higher proportion of cytotoxic T cells (CD3+CD8+). The mucosal treatment with OVA also resulted in a higher proportion of total cells expressing CD27 in the spleen, a marker of memory cells [40]. Although it did not reach statistical significance, this was mainly found on the subset of cells expressing both CD27 and OX12 (a specific marker of B cells). Memory B cells increase during infancy after antigen exposure [41,42] and will rapidly generate immunoglobulins after subsequent exposure which in turn helps infants with pathogen clearance [40].

### 4.3. Effect of a High DHA Maternal Diet on the Establishment of Oral Tolerance in Suckled Pups

We demonstrated for the first time that feeding a maternal diet enriched in DHA during the suckling period differentially affected the *ex vivo* immune response to OVA, a dietary antigen, in a direction that is thought to favor the establishment of OT. Pups from dams fed the high DHA diet during suckling had a higher IL-10 and a lower TGF-β production by OVA-stimulated splenocytes. Although the molecular mechanisms enabling T regulatory cells (Tregs) to modulate OT are not well established, they likely involve the production of TGF-β and IL-10, and the inhibition of IL-2 secretion [8], all of which suppress inflammatory Th1 activity [43]. Therefore, a lower production of TGF-β in response to a dietary antigen in order to maintain a normal proliferative state (*i.e.*, IL-2 production) is suggestive of a more efficient immune response when the maternal diet was supplemented in DHA. IL-10 is a cytokine involved in both cellular and humoral immunity with a primarily anti-inflammatory role and is produced by many immune cells including T cells, B cells and monocytes/macrophages. IL-10 exerts many regulatory properties by suppressing the ability of monocytes/macrophages to produce several pro-inflammatory cytokines (TNF-α and IL-1β) and their ability to induce an exaggerated Th1 response [44,45]. The higher IL-10 production by splenocytes stimulated with either LPS or OVA, suggests that a maternal diet enriched in DHA prevents an exaggerated Th1 response of antigen-presenting cells to a dietary antigen, which prevents a dysregulation of the Th1/Th2 balance. It has also been reported that children at high risk of developing allergies have lower IL-10 production by mononuclear cells stimulated with allergens [46]. Overall, our findings suggest that DHA supplementation in the maternal diet modulates immune function in a direction that is hypothesized to be beneficial for the establishment of OT early in life.

Furthermore, we showed that compared to the control diet, feeding a maternal diet high in DHA also affected the *ex vivo* response to OVA and the tolerance response *in vivo* in non-tolerized pups. Feeding a DHA diet during the suckling period resulted in lower plasma concentrations of OVA-specific IgE antibodies after systemic immunization (IP injection) in non-tolerized pups, similar to the concentrations observed in the tolerized pups. *Ex vivo*, these pups produced more IL-6, even more than cells from the tolerized pups (Figure 3C). Although it did not quite reach statistical significance, similar interactions were also observed in ConA- and LPS-stimulated splenocytes for IL-6 production. This is consistent with our earlier study in which feeding infant formula enriched in AA/DHA resulted in a higher IL-6 production after stimulation with soy (dietary antigen) compared with unsupplemented formula suggesting an increased Th2 response by lymphocytes challenged with dietary antigens [28]. Although the implication of a higher IL-6 production on the establishment of OT is not known, the low plasma concentrations of OVA-specific IgE antibodies after a systemic challenge observed in these untolerized pups fed the high DHA diet during suckling suggest that this might play an important role in mounting tolerance.

This study has strengths and limitations. Although the sample size (N = 12 for the control diet and N = 8 for the DHA diet) is relatively limited, the cross-over design (OVA sensitization) allowed us to have enough statistical power to assess meaningful functional changes in many markers of the immune system. Although using a healthy rat model helps researchers to understand the development of OT in general, it is not necessarily representative of animals or humans at high risk of developing allergic/atopic diseases. This could also be responsible for the overall few differences seen between OVA-tolerized and untolerized pups in cytokines production and plasma Ig concentrations compared to other study [47]. Finally, future experiments should investigate the impact of supplementing the maternal diet in DHA during lactation on the development of OT later in life.

## 5. Conclusions

We showed that, irrespective of the maternal diet, OVA-tolerized pups had a lower Th2 response of antigen-presenting cells combined with a lower Th2 response to a food antigen despite the presence of more activated T cells and memory immune cells. In the context of this healthy rat model, this suggests that when oral tolerance is induced in suckled pups, feeding a maternal diet enriched in DHA does not seem to further affect the ability of splenocytes to respond to different challenges. On the other hand, feeding a maternal diet enriched in DHA during the suckling period improved the ability of splenocytes to respond *ex vivo* to mitogens toward a skewed Th1 response (IFN-γ and TNF-α) but also a skewed Th2 response (IL-6). Supplementing the maternal diet in DHA also changed the response to a dietary antigen (higher IL-10 and lower TGF-β), resulting in a lower concentration of OVA-specific IgE antibodies, suggestive of an overall potential beneficial effect on the establishment of OT in suckled offspring.

## Figures and Tables

**Table 1:** Fatty acid composition of the experimental control and high docosahexaenoic acid (DHA) diet fed to lactating dams adapted from [22] ^a^.

Fatty Acid	Control Diet	DHA Diet
g/100 g of total fatty acids		
C14:0	0.1 ± 0.0	0.4 ± 0.0
C16:0	6.7 ± 0.3	6.2 ± 0.1
C16:1*n*-7	0.2 ± 0.0	0.2 ± 0.1
C18:0	38.8 ± 1.2	40.6 ± 0.2
C18:1*n*-9	29.0 ± 1.7	24.8 ± 0.3
C18:2*n*-6	21.2 ± 0.5	21.6 ± 0.0
C20:0	0.9 ± 0.0	0.9 ± 0.0
C18:3*n*-3 (ALA)	1.7 ± 0.1	3.3 ± 0.1
C20:3n-6	0.4 ± 0.1	0.4 ± 0.1
C20:4*n*-6 (AA)	0.4 ± 0.0	0.4 ± 0.0
C22:6*n*-3 (DHA)	0	0.9 ± 0.1
Other fatty acids ^b^	0.8	0.4
Total SFA	46.5 ± 0.8	48.1 ± 0.3
Total PUFA	23.6 ± 0.6	26.6 ± 0.1
Total *n*-6	21.9 ± 0.5	22.3 ± 0.1
Total *n*-3	1.6 ± 0.1	4.2 ± 0.1
Total MUFA	29.1 ± 1.7	24.9 ± 0.4
Ratio *n*-6/*n*-3	13.3	5.3
Ratio PUFA/SFA	0.5	0.6

Notes: ^a^ Analysis by GLC of *n* = 2 batches, mean ± SEM; AA, arachidonic acid; ALA, α-linolenic acid; DHA, docosahexaenoic acid; MUFA, monounsaturated fatty acids; n, omega; PUFA, polyunsaturated fatty acids; SFA, saturated fatty acids; ^b^ other fatty acids refer to fatty acids that contributed for less than 0.1% in the diet which included trace of 10:0, 12:0, 20:2*n*-6, 20:5*n*-3, 22:0, 22:4*n*-6 and 22:5*n*-3.

**Table 2:** Effect of the control and the high DHA diet fed during the suckling period on the *ex vivo* cytokine production of stimulated splenocytes by mitogens or ovalbumin of three-week pups according to the oral tolerance treatment ^a^.

Variable	Control Diet		DHA Diet		*P* ^b^	*P* ^c^	*P* ^inter^
	Placebo Treatment (N = 12)	OVA Treatment (N = 11)	Placebo Treatment (N = 8)	OVA Treatment (N = 8)			
*Con A*							
IL-2, *pg/mL*	4032 ± 240	4069 ± 346	4294 ± 306	3954 ± 260	0.782	0.576	0.539
IL-6, *pg/mL*	545 ± 81	589 ± 114	949 ± 214	738 ± 126	0.136	0.212	0.062
IL-10, *pg/mL*	517 ± 41	484 ± 54	654 ± 83	542 ± 69	0.137	0.205	0.364
IFN-γ, *pg/mL*	311 ± 53	354 ± 68	769 ± 190	653 ± 159	0.045	0.584	0.385
TNF-α, *pg/mL*	111 ± 12	110 ± 11	139 ± 14	125 ± 11	0.217	0.457	0.552
*LPS*							
IL-1β, *pg/mL*	265 ± 39	265 ± 41	350 ± 65	335 ± 61	0.216	0.762	0.924
IL-6, *pg/mL*	1463 ± 221	1310 ± 161	2362 ± 208	1439 ± 118	0.043	0.011	0.064
IL-10, *pg/mL*	274 ± 17	218 ± 14	350 ± 48	346 ± 35 ^b^	0.010	0.364	0.436
IFN-γ, *pg/mL*	73 ± 18	43 ± 9	81 ± 31	105 ± 53	0.462	0.936	0.251
TNF-α, *pg/mL*	1433 ± 180	1434 ± 201	2039 ± 169	2027 ± 243	0.036	0.954	0.847
*OVA*							
IL-2, *pg/mL*	18 ± 0	18 ± 1	18 ± 1	20 ± 1	0.272	0.222	0.409
IL-6, *pg/mL*	716 ± 74	788 ± 87	1038 ± 188	788 ± 111	0.254	0.170	0.035
IL-10, *pg/mL*	179 ± 16	176 ± 21	277 ± 28	237 ± 23	0.012	0.373	0.224
IL-13, *pg/mL*	34 ± 4	19 ± 2	32 ± 2	18 ± 3	0.654	<0.001	0.859
TGF-β, *pg/mL*	1262 ± 83	1257 ± 51	1010 ± 68	958 ± 47	0.001	0.687	0.740

Notes: ^a^ Values are presented as mean ± SEM; ConA, Concanavalin A; DHA, docosahexaenoic acid; IFN, interferon; IL, interleukin; LPS, lipopolysaccharide; OVA, ovalbumin; TGF, transforming growth factor; TNF, tumor necrosis factor; ^b^ *p* value from the main effect of the **suckling diet** in the Mixed model on three-week pups’ outcomes; ^c^ *p* value from the main effect of the **oral tolerance treatment** in the Mixed model on three-week pups’ outcomes. *P*^inter^ *p* interaction between the suckling diet and the oral tolerance treatment in the Mixed model on three-week pups’ outcomes.

**Table 3:** Splenocyte phenotypes of three-week pups fed the control and the high DHA diet during the suckling period according to the oral tolerance treatment ^a^.

Variable	Control Diet		DHA Diet		*P ^b^*	*P ^c^*	*P* ^inter^
	Placebo Treatment (N = 6)	OVA Treatment (N = 6)	Placebo Treatment (N = 6)	OVA Treatment (N = 6)			
*% of total cells*							
Total CD3+	20.9 ± 1.2	22.5 ± 1.0	22.1 ± 0.5	23.2 ± 0.9	0.464	0.010	0.443
CD3+CD4+ (helper T cells)	10.7 ± 0.7	11.6 ± 0.2	11.3 ± 0.4	11.7 ± 0.4	0.587	0.186	0.578
CD3+CD8+ (cytotoxic T cells)	6.5 ± 0.2	7.3 ± 0.5	7.1 ± 0.3	8.2 ± 0.4	0.118	0.003	0.800
% of CD3+ cells that also express CD4+	51.1 ± 0.5	49.7 ± 2.1	50.6 ± 0.3	45.9 ± 2.1	0.194	0.068	0.298
% of CD3+ cells that also express CD8+	30.5 ± 1.1	33.0 ± 0.5	30.8 ± 0.9	34.5 ± 1.0	0.354	0.004	0.510
Ratio CD4/CD8	2.1 ± 0.2	1.9 ± 0.2	2.0 ± 0.1	1.8 ± 0.0	0.419	0.079	0.757
Total CD28+	37.1 ± 1.9	39.4 ± 1.9	38.9 ± 2.4	44.4 ± 2.7	0.190	0.016	0.586
% of CD3+ cells that also express CD8+ CD28+	31.2 ± 1.3	34.1 ± 1.0	29.7 ± 0.7	34.7 ± 0.9	0.696	0.002	0.323
Total OX62+	9.2 ± 0.5	10.6 ± 0.3	9.0 ± 0.5	11.4 ± 1.3	0.676	0.020	0.508
CD3+OX62+	4.6 ± 0.4	5.8 ± 0.3	5.0 ± 0.2	5.3 ± 0.2	0.879	0.004	0.128
CD3+CD4+CD25+FoxP3+ (Treg)	8.9 ± 1.1	9.3 ± 0.7	8.3 ± 0.7	7.8 ± 0.5	0.178	0.940	0.427
Total CD27+	42.7 ± 0.8	44.1 ± 0.7	41.5 ± 1.4	42.7 ± 1.2	0.299	0.003	0.104
OX12+CD27+	15.0 ± 1.5	15.9 ± 1.6	13.4 ± 0.9	14.6 ± 1.1	0.639	0.108	0.636
OX62+CD74+ (dentritic cells)	7.3 ± 0.3	6.9 ± 0.4	6.4 ± 0.2	6.7 ± 0.4	0.120	0.966	0.281

Notes: ^a^ Values are presented as mean ± SEM; Values are a proportion of the total gated cells as determined by immunofluorescence. CD, cluster of differentiation; DHA, docosahexaenoic acid; No significant differences were observed in any other phenotypes between groups. No significant differences were observed among groups (N = 24; mean ± SEM) for total cells expressing CD25 (8.2 ± 0.3), CD152 (6.9 ± 0.5), CD68 (macrophages, 11.0 ± 0.8), CD284 (10.9 ± 0.7), IgG (4.7 ± 0.4), IgM (52.7 ± 2.4) and IgA (12.9 ± 0.8) or CD4+CD25+ (3.9 ± 0.4), CD4+CD27+ (13.4 ± 0.4), CD4+CD152+ (2.7 ± 0.3) and CD68+CD284+ (6.7 ± 0.5) cells; ^b^ *p* value from the main effect of the suckling diet in the Mixed model on three-week pups’ outcomes; ^c^ *p* value from the main effect of the oral tolerance treatment in the Mixed model on three-week pups’ outcomes. *P*^inter^ *p* interaction between the suckling diet and the oral tolerance treatment in the Mixed model on three-week pups’ outcomes.

## Supplementary Materials

The following are available online at http://www.mdpi.com/2072-6643/8/2/103/s1, Table S1: Effect of the control and the high DHA diet fed during the suckling period on growth parameters of three-week pups according to the oral tolerance treatment; Table S2: Mesenteric lymphocyte phenotypes of three-week pups fed the control and the high DHA diet during the suckling period according to the oral tolerance treatment.

## Abbreviations

The following abbreviations are used in this manuscript:

AA: arachidonic acid
ALA: α-linolenic acid
ConA: Concanavalin A
DHA: docosahexaenoic acid
DPA: docosapentaenoic acid
IFN-γ: interferon-gamma
Ig: immunoglobulin
IL: interleukin
LPS: lipopolysaccharide
MUFA: monounsaturated fatty acid
n: omega
OVA: ovalbumin
PUFA: polyunsaturated fatty acid
SFA: saturated fatty acid
TGF-β: transforming growth factor-beta
TNF-α: tumor necrosis factor-alpha
