# Supplementary material for: Feeding a Diet Enriched in Docosahexaenoic Acid to Lactating Dams Improves the Tolerance Response to Egg Protein in Suckled Pups
Source: Nutrients. 2016 Feb 19;8(2):103. doi: 10.3390/nu8020103 (PMC4772065; doi:10.3390/nu8020103)
Supplement: Supplementary file 1 [file nutrients-08-00103-s001.doc]

**Supplementary Materials: Feeding a Diet Enriched
in Docosahexaenoic Acid to Lactating Dams Improves the Tolerance Response to Egg
Protein in Suckled Pups**

Caroline Richard, Erin D. Lewis, Susan Goruk and Catherine J. Field

**Table S1.** Effect of the control and the high DHA diet fed during the suckling period on growth parameters of 3 weeks pups according to the oral tolerance treatment ^a^.

| **Variable** | **Control Diet** | | **DHA Diet** | | ***P ^b^*** | ***P ^c^*** | ***P* ^inter^** |
| --- | --- | --- | --- | --- | --- | --- | --- |
|  | **Placebo Treatment (N = 12)** | **OVA Treatment (N = 12)** | **Placebo Treatment (N = 8)** | **OVA Treatment (N = 8)** |  |  |  |
| Body weight (g) | 50.8 ± 1.2 | 50.3 ± 1.9 | 50.1 ± 3.1 | 50.2 ± 3.0 | 0.873 | 0.872 | 0.494 |
| Spleen weight (g) | 0.3 ± 0.0 | 0.3 ± 0.0 | 0.3 ± 0.0 | 0.3 ± 0.0 | 0.508 | 0.481 | 0.089 |
| Liver weight (g) | 2.1 ± 0.1 | 2.2 ± 0.1 | 2.4 ± 0.1 | 2.4 ± 0.1 | 0.086 | 0.558 | 0.594 |
| Gut length (cm) | 68.4 ± 1.2 | 68.7 ± 1.5 | 71.9 ± 2.1 | 71.8 ± 1.6 | 0.119 | 0.920 | 0.867 |
| Splenocytes (10^6^/g spleen) | 39.1 ± 3.2 | 39.2 ± 2.9 | 39.0 ± 6.8 | 44.8 ± 7.1 | 0.582 | 0.488 | 0.497 |

Notes: ^a^ Values are presented as mean ± SEM; DHA, docosahexaenoic acid; ^b^ *p* value from the main effect of the **suckling diet** in the Mixed model on 3 weeks pups’ outcome; ^c^ *p* value from the main effect of the **oral tolerance treatment** in the Mixed model on 3 weeks pups’ outcome. *P*^inter^ *p* interaction between the suckling diet and the oral tolerance treatment in the Mixed model on
3 weeks pups’ outcome.

**Table S2.** Mesenteric lymphocyte phenotypes of 3 weeks pups fed the control and the high DHA diet during the suckling period according to the oral tolerance treatment ^a^.

| **Variable** | **Control Diet** | | **DHA Diet** | | ***P ^b^*** | ***P ^c^*** | ***P* ^inter^** |
| --- | --- | --- | --- | --- | --- | --- | --- |
|  | **Placebo Treatment (N = 5)** | **OVA Treatment (N = 5)** | **Placebo Treatment (N = 5)** | **OVA Treatment (N = 7)** |  |  |  |
| ***% of Total Cells*** | | | | | | | |
| Total CD3+ | 68.9 ± 1.3 | 72.0 ± 1.3 | 72.5 ± 0.9 | 72.7 ± 1.4 | 0.136 | 0.169 | 0.217 |
| Total CD4+ | 52.9 ± 1.5 | 53.4 ± 0.9 | 55.7 ± 1.1 | 55.2 ± 0.7 | 0.125 | 0.562 | 0.324 |
| Total CD8+ | 21.5 ± 0.3 | 24.5 ± 0.9 | 22.6 ± 0.6 | 23.7 ± 0.8 | 0.840 | **0.011** | 0.119 |
| CD3+CD4+ (helper T cells) | 51.1 ± 1.5 | 50.8 ± 0.9 | 53.8 ± 0.9 | 52.9 ± 1.1 | 0.072 | 0.651 | 0.803 |
| CD3+CD8+ (cytotoxic T cells) | 18.4 ± 1.3 | 21.4 ± 1.4 | 17.2 ± 0.4 | 18.3 ± 0.8 | 0.096 | 0.059 | 0.303 |
| Ratio CD4/CD8 | 2.5 ± 0.1 | 2.2 ± 0.1 | 2.5 ± 1.0 | 2.4 ± 0.1 | 0.307 | **0.019** | 0.335 |
| Total CD28+ | 71.4 ± 1.4 | 74.3 ± 0.9 | 69.4 ± 2.2 | 70.2 ± 1.7 | 0.099 | 0.186 | 0.361 |
| CD4+CD28+ | 51.6 ± 1.6 | 52.4 ± 0.9 | 52.4 ± 1.4 | 52.1 ± 1.1 | 0.855 | 0.863 | 0.673 |
| CD8+CD28+ | 17.5 ± 0.4 | 20.2 ± 0.8 | 15.8 ± 1.0 | 16.6 ± 0.8 | **0.014** | **0.034** | 0.135 |

Notes: ^a^ Values are presented as mean ± SEM; Significant differences are indicated in bold; Values are a proportion of the total gated cells as determined by immunofluorescence. CD, cluster of differentiation; DHA, docosahexaenoic acid; No significant differences were observed among groups (N = 22; mean ± SEM) for total cells expressing CD25 (8.2 ± 0.6), CD27 (51.3 ± 2.0) and CD152 (4.5 ± 3.2) or CD4+CD25+ (6.5 ± 0.4), CD8+CD25+ (2.2 ± 0.2), CD4+CD27+ (37.8 ± 1.6), CD8+CD27+ (14.6 ± 0.8), CD4+CD152+ (3.3 ± 2.4), CD8+CD152+ (1.1 ± 0.8) cells; ^b^ *p* value from the main effect of the **suckling diet** in the Mixed model on 3 weeks pups’ outcome; ^c^ *p* value from the main effect of the **oral tolerance treatment** in the Mixed model on 3 weeks pups’ outcome. *P*^inter^ *p* interaction between the suckling diet and the oral tolerance treatment in the Mixed model on 3 weeks
pups’ outcome.
